# The neuroprotective effect of pretreatment with carbon dots from *Crinis Carbonisatus* (carbonized human hair) against cerebral ischemia reperfusion injury

**DOI:** 10.1186/s12951-021-00908-2

**Published:** 2021-08-28

**Authors:** Yue Zhang, Suna Wang, Fang Lu, Meiling Zhang, Hui Kong, Jinjun Cheng, Juan Luo, Yan Zhao, Huihua Qu

**Affiliations:** 1 School of Life Science, Beijing University of Chinese Medicine, Beijing, China; 2 School of Basic Medical Sciences, Beijing University of Chinese Medicine, 11 Beisanhuandong Road, Chaoyang District, Beijing, 100029 China; 3 School of Basic Medical Sciences, Guizhou University of Chinese Medicine, Beijing, China; 4 Center of Scientific Experiment, Beijing University of Chinese Medicine, 11 Beisanhuandong Road, Chaoyang District, Beijing, 100029 China

**Keywords:** *Crinis Carbonisatus*, Carbon dots, Neuroprotective effect, Ischemic stroke, Cerebral ischemia reperfusion

## Abstract

**Background:**

Cerebral infarction and cerebral hemorrhage, also known as “stroke”, is one of the leading cause of death. At present, there is no real specific medicine for stroke. *Crinis Carbonisatus* (named Xue-yu-tan in Chinese), produced from carbonized hair of healthy human, and has been widely applied to relieve pain and treat epilepsy, stroke and other diseases in China for thousands of years.

**Results:**

In this work, a new species of carbon dots derived from *Crinis Carbonisatus* (CrCi-CDs) were separated and identified. And the neuroprotective effect of carbon dots from CrCi were evaluated using the middle cerebral artery occlusion (MCAO) model. Neurological deficit score and infarction volume was assessed, evans blue content of ischemic hemispheres was measured, the concentrations of inflammatory factors, tumor necrosis factor-α (TNF-α), interleukin-6 (IL-6) in the cortex were measured, and the levels of neurotransmitters in the brain were determined. Preconditioning of CrCi-CDs significantly reduced ischemic lesion volume and blood–brain-barrier (BBB) permeability, improved neurologic deficits, decreased the level of TNF-α and IL-6 in MCAO rats, inhibited excitatory neurotransmitters aspartate (Asp) and glutamate (Glu), and increased the level of 5-hydroxytryptamine (5-HT). The RNA-Sequencing results reveal that further potential mechanisms behind the activities may be related to the anti-inflammation effects and inhibition of neuroexcitatory toxicity.

**Conclusion:**

CrCi-CDs performs neuroprotective effect on cerebral ischemia and reperfusion injury, and the mechanisms may correlate with its anti-inflammatory action, which suggested that CrCi-CDs have potential value in clinical therapy on the acute apoplexy cases in combination with thrombolytic drugs.

**Graphic abstract:**

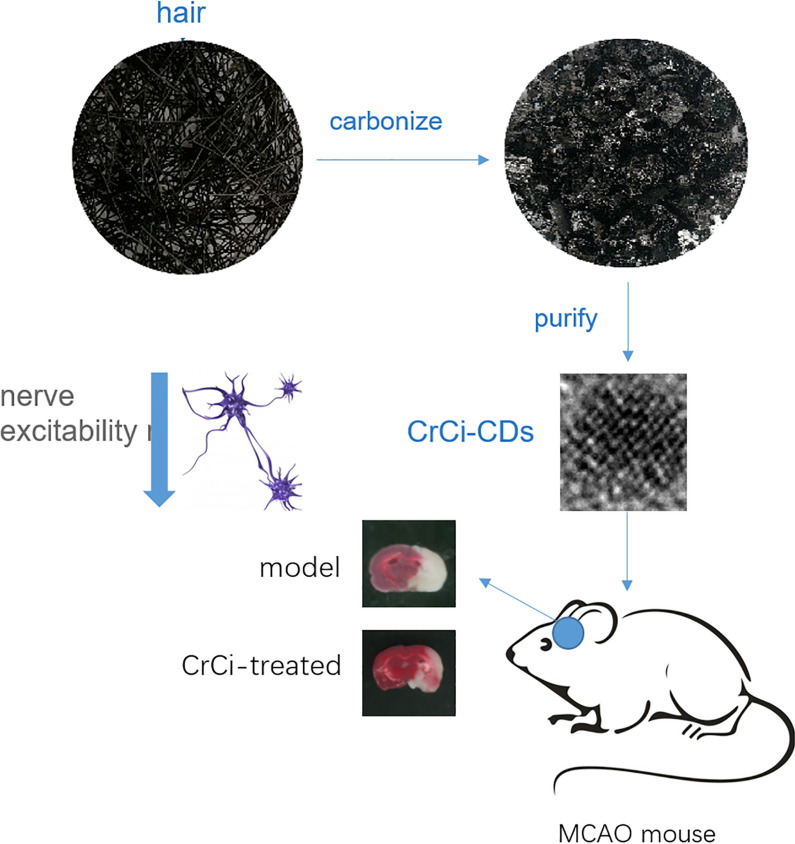

**Supplementary Information:**

The online version contains supplementary material available at 10.1186/s12951-021-00908-2.

## Background

Cerebral infarction and cerebral hemorrhage, also known as “stroke”, is the first leading cause of death in China [1] and one of the top three causes of death in many countries [2]. Stroke is mainly divided into ischemic stroke and hemorrhagic stroke, ischemic stroke accounts for the majority. Ischemic stroke is a common and frequently-occurring disease, has high morbidity, mortality, disability and relapse rate, brings not only physical and mental suffering to the patients, but also places a huge burden on families and society.

An ischemic stroke may occur when blood flow to some part of the brain is blocked. The block is usually due to atherosclerosis or a blood clot clogs up a narrow blood vessel. When oxygen cannot get to an area of the brain, tissue in that area may get damaged [3]. A lot of evidence has shown that it’s not just ischemia that causes tissue damage [4], when restore the blood supply, the excess free radicals and extensive inflammatory response resulted to reperfusion injury [5].

The ischemic reperfusion injury is a complex pathophysiological, associates with the release of excitatory amino acids, ion disequilibrium, apoptosis, necrosis, oxidative stress, and inflammation [6]. Especially, reactive oxygen species (ROS) and inflammation plays a major role in the process of injury, amplify and propagate neuronal damage following ischemic central nervous system (CNS) injury [7]. Therefore, therapeutic approaches enhancing ischemia tolerance have critical clinical application value and could be used in a preventive manner during an intervention for reperfusion injury [8]. In clinical practice, the second-level prevention (pre-symptomatic) administration was recommended for the people at risk of stroke. Nevertheless, there is still lack of safe and effective therapeutic strategy of precondition for ischemic stroke, it is essential to establish a novel preventive strategy for ischemic stroke.

However, to some extent, there is no real specific medicine for stroke. At present, intravenous thrombolysis via recombinant tissue plasminogen activator (rtPA) is the only pharmacological treatment for acute ischemic stroke approved by US Food and Drug Administration (FDA) [9]. But it is limited by the short treatment time window and the potential for hemorrhagic transformation. And vascular recanalization caused the damage which called ischemia reperfusion injury is inevitable.

Current therapeutic approaches of neurogenic disease are symptomatic treatment and of modest efficacy. Recently, nanotechnology bring innovations to the fields of medicine and pharmacology [10, 11]. The medical and pharmacological applications of carbon-based nanoparticles (such as carbon nanotubes and graphene) have received much attention due to their unique physicochemical properties [12]. For instance, amine-modified single-walled carbon nanotubes were proved to be effective to protect neurons from injury in a rat stroke model [13]. And researchers found that single-walled carbon nanotubes can alter cytochrome-c electron transfer and modulate mitochondrial function [14]. Moreover, aggregated single-walled carbon nanotubes attenuate the behavioural and neurochemical effects of methamphetamine in mice [15].

Moreover, several carbon-based nanoparticles showed neuroprotective effect, the main mechanisms including anti-inflammatory, reduce the ROS, autophagy regulation and immunoregulation [16]. For instance, nanodiamond was indicated to have neuroprotective effect in Alzheimer’s Disease (AD) by modulating NF-κB and STAT3 signaling [17]. Graphene oxide (GO) can reduce oxidative stress and inhibit neurotoxicity through catalase-like activity [18]. GO can ameliorate the cognitive impairment in AD through inhibiting PI3K/Akt/mTOR pathway to induce autophagy [19]. GO also enhances β-amyloid clearance by inducing autophagy of microglia and neurons [20]. Moreover, graphene quantum dots were reported to prevent α-synucleinopathy in Parkinson’s disease [21], and improve learning in AD [22]. These researches predicted that carbon-based nanoparticles can make a big difference in the field of neurotherapy.

Carbon dots (CDs), discovered in discharge soot in 2004 [23], defined by characteristic sizes of ˂ 10 nm, containing some surface passivation [24], have aroused the growing concern in carbon nanomaterials [25]. CDs exhibit unique structural and electronic properties making them a promising platform for diverse applications including imaging, biosensors [26], catalytic, energy and biomedical use. In our previous work, several kinds of CDs were identified from carbonized medical nature products, which have various effects such as immune regulation, anti-inflammation and hemostasis (Additional file 1: Table S1). However, the large precursor compositions may lead to heterogeneity in natural products-derived CDs [27]. Hence, CDs with definite chemical structures and controlled morphology are being pursued [28, 29].

*Crinis Carbonisatus* (CrCi), named Xue-yu-tan in Chinese, is a traditional Chinese medicinal materials. It is produced from the carbonized hair of healthy human, after washing, drying, and calcining human hair. CrCi is widely used and has a long-term medical history in China. It was first recorded in the *Prescriptions for Fifty-two Diseases* more than 2000 years ago. Classic Chinese medical books documented the *Crinis Carbonisatus* could be used to treat various diseases, such as hemorrhage, epilepsy, and stroke.

After observed the extensive folk application and magical curative effect of CrCi, we wonder if it has a definite effect, and its mechanism have not been clearly explained. Recently, we discover and purify carbon dots (CDs) from the water extract of CrCi (CrCi-CDs). We speculated that the novel nanomaterial with electron transfer ability and versatile surface groups may be an active pharmaceutical ingredient related to Xue-Yu-Tan’s neuroprotective effect.

Hence, in this study, we used an ischemia–reperfusion injury model of stroke to explore whether rats that received CrCi-CDs preoperatively could be ameliorated blood brain barrier (BBB) destruction and protected from cerebral ischemia reperfusion injury. We assessed the therapeutic effects of CrCi-CDs by measuring neurobehavioral functions and infarct volumes, assessing of BBB permeability, analyzing the IL-6, TNF-α levels, observing the histological and ultrastructural changes of brain tissue by Hematoxylin–Eosin Stain (HE staining) and Transmission electron microscope (TEM), and measuring the contents of brain monoamine neurotransmitters.

## Results

### Characterization of CrCi-CDs

**Fig. 1:**
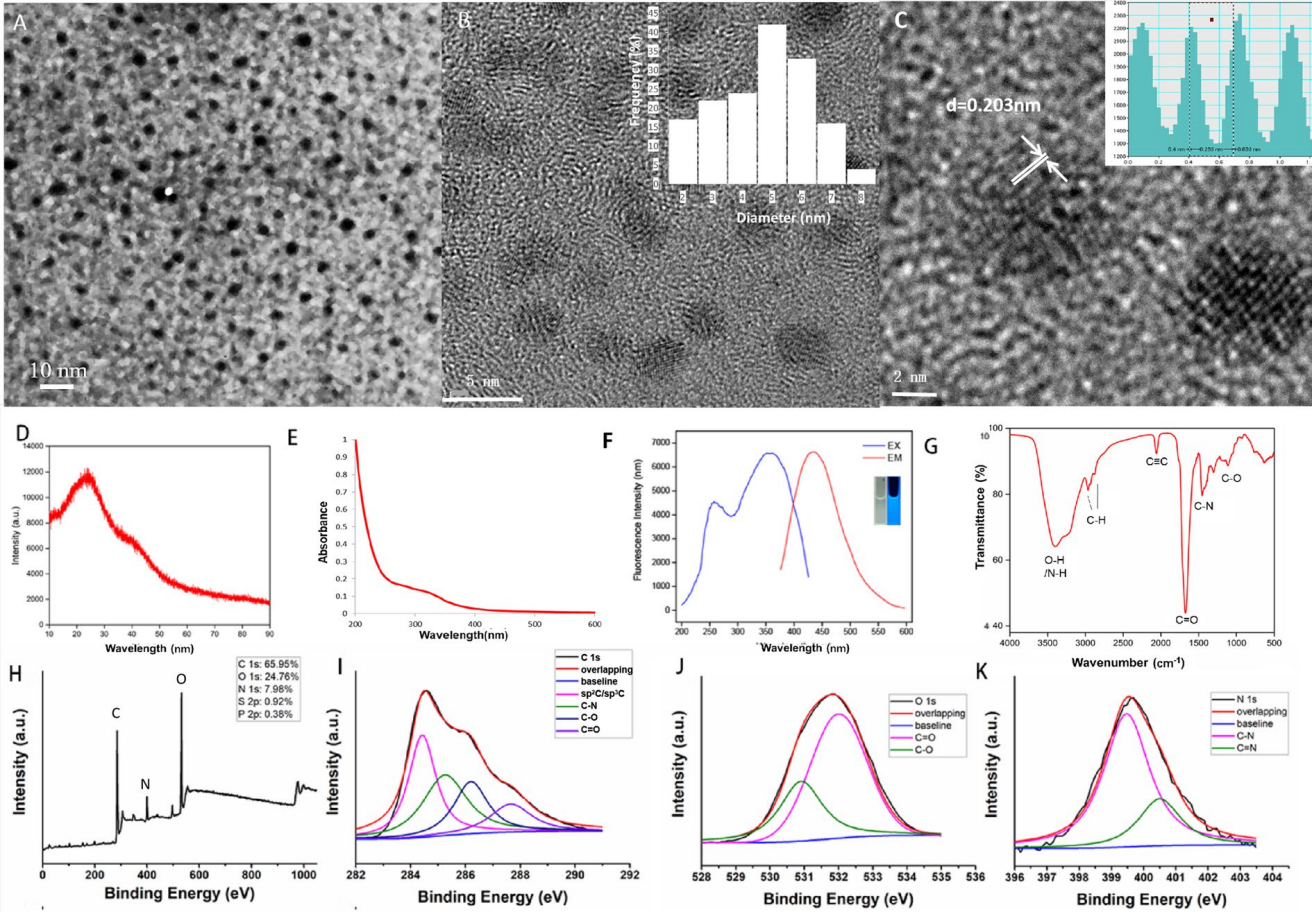
**A** Transmission electron microscopy (TEM) image of carbon dots from *Crinis Carbonisatus* (CrCi-CDs). (magnification ×160,000), the accelerating voltage was 100 kV. **B**, **C** High-resolution TEM (HRTEM) images of individual CrCi-CDs. **E** UV–visible spectra of CrCi-CDs. **F** Emission spectra Excitation spectra of CrCi-CDs with different emission wavelengths of CrCi-CDs excited at 360 nm. **G** Fourier transform infrared (FTIR) spectrum of the CrCi-CDs (32 scans at 2 cm^−1^ resolution in the scanning range of 500–4000 cm^−1^). **H**–**K** Elemental analysis of CrCi-CDs

As shown in Fig. 1A, CrCi-CDs were observed spherical and uniformly distributed by TEM. Diameter range is 3.2–8.8 nm, the average particle size was less than 10 nm (Fig. 1B). The lattice spacing of CrCi-CDs was 0.203 nm (Fig. 1C). The X-Ray Diffraction (XRD) pattern of CrCi-CDs displays a typical single broad peak at around 24.5 (shown in Fig. 1D), which is consistent with that of the plane of carbon dots that previously reported.

As shown in Fig. 1E, the UV–Vis spectrum of the CrCi-CDs showed a weak and broad adsorption around 260 nm to 340 nm, which can be ascribed to the π–π* transition of the aromatic C=C/C≡C bond and n–π* electronic transitions of the heteroatom such as C=O.

Fig. 1F showed the fluorescence excitation and emission spectrum of CrCi-CDs aqueous solution. Under the excitation wavelength of 361 nm, the emission peak of CrCi-CDs is about 435 nm. The solution of CrCi-CDs gave off bright blue fluorescence when irradiated by a 365 nm ultraviolet lamp. In this study, the quantum yield of CrCi-CDs was calculated to be 4.03%.

In the FTIR spectrum of the CrCi-CDs (Fig. 1G), two peaks located at 2920 cm^−1^ and 2860 cm^−1^ indicated sp^3^ and sp^2^ C–H stretching vibrations of –CH3 and –CH2. The peak at 3500–3200 cm^−1^ was attributed to the stretching vibration of O–H and N–H bonds. Absorption at 1340 cm^−1^ can be identified as amino C–N stretching vibration. Peaks at 1625 cm^−1^ and 1050 cm^−1^ may correspond to the existence of C=O and C–O, respectively. The peak at 2200 cm^−1^ implied that the existence of sp hybridization carbons in CrCi-CDs. Therefore, we concluded that the CrCi-CDs possesses abundant hydroxyl, amino and carbonyl/carboxylate groups at their surfaces.

Elemental analysis (Fig. 1H) by XPS spectra (X-ray photoelectron spectroscopy) showed the three major elements, including C (65.95%), O (24.76%), and N (7.98%). These results in Fig. 1I–K suggested the elements C, O, and N might correspond to C–C, C=C, C=O, C–N and C–O bonds. The XPS result was in accordance with the surface composition of the CrCi-CDs shown in the FTIR analysis.

### CrCi-CDs reduced brain infarction in MCAO model rats

**Fig. 2:**
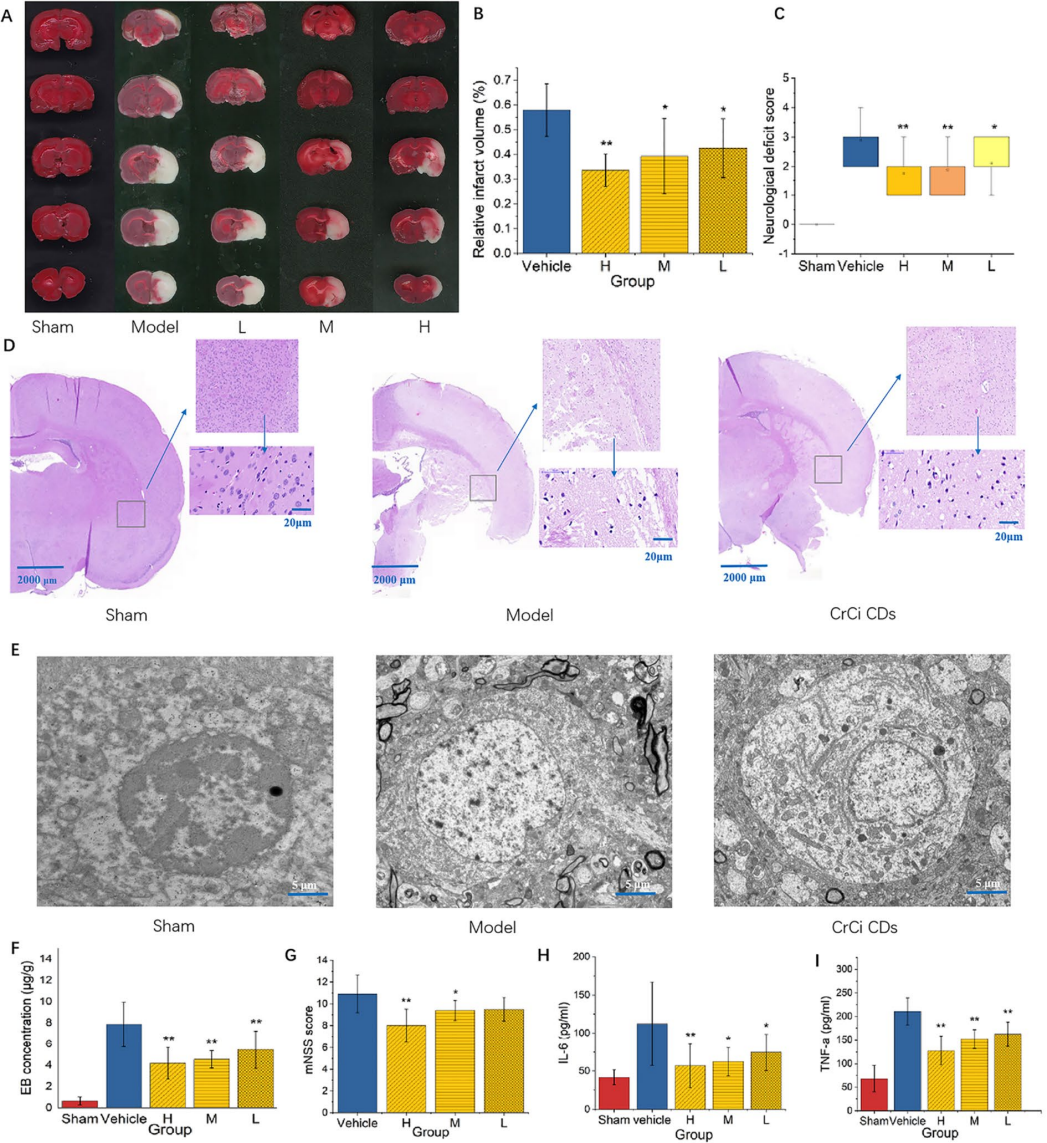
Effects of CrCi-CDs on neurological deficit scores, infarct volume. **A** Representative TTC staining of brain slices in rats, 24 h after MCAO. Neurologic deficit scores are presented as the median (range), were evaluated at 24 h after MCAO. *p < 0.05, **p < 0.01 versus vehicle group, n = 18. **B** Statistical analysis of the percentage of infarct volume was determined, 24 h after MCAO. **C** Effects of CrCi-CDs on neurological deficit scores. **D** Brain photos and brain slices with HE stains. **E** The ultra-structure of neuron in cortex, EM × 5 k, 20 kA. **F** Permeability of BBB as revealed by evans blue staining. **G** Effects of CrCi-CDs on mNSS score. Bar graph shows the levels of IL-6 (**H**), and TNF-α (**I**) in the cortex of the ischemic hemisphere 24 h after reperfusion

To evaluate the effect of CrCi-CDs on area of cerebral infarction, a standard TTC stain test was carried out. The brain tissues was stained with TTC for 24 h after MCAO. As shown in Fig. 2A, the viable cerebral tissue was stained red while the infarcted cerebral tissue remained pale, no infarction area was observed in sham rats. Figure 2B shows the effects of CrCi-CDs pretreatment on infarcted volume induced by MCAO. Compared with vehicle group (57.9% ± 10.6%), the cerebral infarction area in low dosage CrCi-CDs pretreated group (1 mg/kg) and medium dosage (2 mg/kg) group, were significantly decreased (42.5% ± 11.9%, 39.3% ± 15.2%, P < 0.05) respectively. And the high dosage group (4 mg/kg) was notably decreased 33.6% ± 6.6%. (P < 0.01, Fig. 2B).

### Improved neurological function

To evaluate the therapeutic effects of CrCi-CDs, a 0 to 4 point scale was applied to evaluate neurological function. The neurological deficit score of the vehicle group was obviously higher than the sham group. Rats pretreated with CrCi-CDs (1 mg/kg) resulted in a decrease in neurological scores in comparison with the vehicle group (2.89 ± 0.68 for the vehicle group, 2.11 ± 0.68 for the 1 mg/kg CrCi-CDs group, P < 0.05, Fig. 2C). Rats pretreated with CrCi-CDs at the dose of (2, 4 mg/kg) exhibited significantly less neurological dysfunction compared to those pretreated with vehicle (1.72 ± 0.58 for the 4 mg/kg CrCi-CDs group, 1.89 ± 0.68 for the 2 mg/kg CrCi-CDs group, P < 0.01, Fig. 2C).

### Histopathological analysis

As shown in Fig. 2D, in the sham group, no significant change was observed in the morphology and structure of glial cells, neurons, and capillaries in the brain tissue, and the nuclear structure was intact and clearly visible. In the vehicle group, optical microscopes observation revealed the evident edema and extensive vacuolization in brain tissue. Necrosis, coagulated and disordered neurons of the cerebral cortex were identified. The tissue changes also including dropsy indifferent degree, inflammatory cell infiltrate and gliocyte hyperplasia. Compared with the model group, the degree injury of brain tissue in the CrCi-CDs groups was significantly reduced, the vacuoles in brain tissue space were less, and fixed shrinkage were also significantly reduced.

### TEM analysis

Electronmicroscope observation revealed many electron dense granulation and vacuolus in the rat brain of focal cerebral ischemia and reperfusion (vehicle group). Neurons were swollen, various membrane structures were dissolved, broken and discontinuous. Fractured cristae and vacuolization were observed in the mitochondria. While, the ultrastructural damage of neurons were significantly decreased in CrCi-CDs groups (Fig. 2E).

The HE staining (Fig. 2D) and electromicroscope (Fig. 2E) results indicate the brain tissue integrity, inflammatory exudation and edema degree of CrCi-CDs groups is obviously improved than model group and approached normal group. However, we do not observe the CDs in brain tissue.

### CrCi-CDs regulates BBB permeability after cerebral ischemia

To evaluate BBB permeability after ischemic injury, BBB leakage was measured using evans blue (EB) extravasation assay. As shown in Fig. 2F, EB concentration of the ischemic hemisphere in vehicle group (7.84 ± 2.1 µg/g) and CrCi-CDs treated groups (4.18 ± 1.48 µg/g for the 4 mg/kg CrCi-CDs group, 4.54 ± 0.82 µg/g for the 2 mg/kg group, 5.46 ± 1.76 µg/g for the 1 mg/kg group) were significantly greater than that of sham group (0.626 ± 0.37 μg/g, P < 0.01). These results indicated that EB extravasation was increased in ischemic rats, indicating the disruption of BBB. EB content in the ischemic hemisphere was significantly decreased in all dosage of CrCi-CDs pretreated groups, compared with the vehicle group. There was no statistically significant difference in EB concentrations in the right brain hemispheres between different dosages of CrCi-CDs groups. The above results suggested that pretreatment with CrCi-CDs (1, 2, 4 mg/kg) decreased BBB leakage.

### Neurological function

Neurological function was evaluated by a scale of a 0 to 4 point (normal score, 0; maximal deficit score, 44). Results showed that neurological severity score (mNSS) score of the rats in all dosages of CrCi-CDs groups significantly reduced, compared with the vehicle group (Fig. 2G).

### CrCi-CDs suppresses MCAO-induced inflammation

To examine whether CrCi-CDs were involved in the inflammatory response in ischemic brain injury, inflammatory mediators including IL‑6 and TNF‑α in the cortex of the ischemic hemisphere were detected.

Rats pretreated with CrCi-CDs (1, 2 mg/kg) had decreased IL-6 concentration in the cortex of the ischemic hemisphere than those in the vehicle group (112.27 ± 54.79 pg/mL for the vehicle group, 62.06 ± 18.69 pg/mL for the 2 mg/kg group, 74.53 ± 23.84 pg/mL for the 1 mg/kg group, P < 0.05, Fig. 2H). Moreover, rats pretreated with a high dose of CrCi-CDs (4 mg/kg) remarkably decreased IL-6 level in the cortex of the ischemic hemisphere compared with the vehicle group (56.87 ± 28.81 pg/mL for the 4 mg/kg CrCi-CDs group, P < 0.01, Fig. 2H).

In comparison with the vehicle group (210.32 ± 28.56 pg/m), CrCi-CDs pretreatment (1 mg/kg, 2 mg/kg, 4 mg/kg) significantly decreased TNF-α concentration in the cortex of the ischemic hemisphere (127.76 ± 29.78 pg/mL for the 4 mg/kg CrCi-CDs group, 151.96 ± 19.77 pg/mL for the 2 mg/kg group, 162.07 ± 25.41 pg/mL for the 1 mg/kg group P < 0.01, Fig. 2I).

### Effects of CrCi-CDs on neurotransmitter content in MCAO rats

**Fig. 3:**
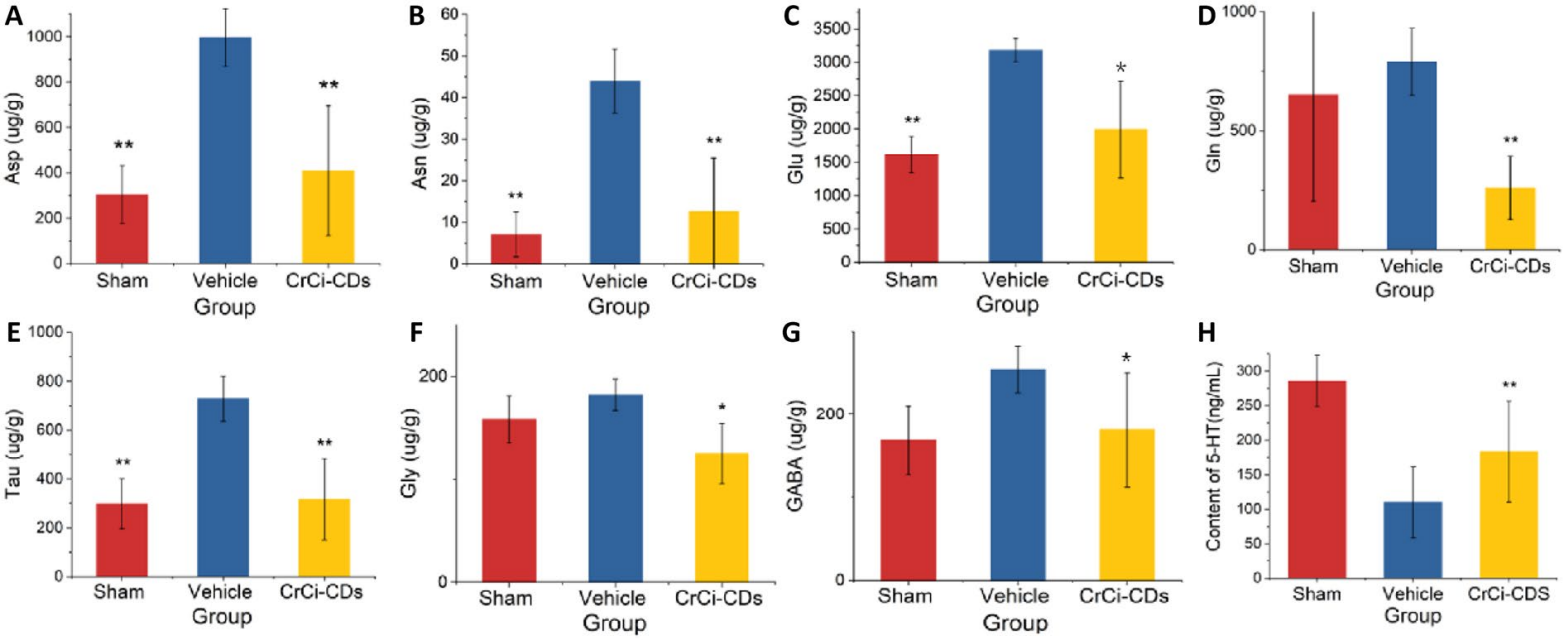
Changes in amino acid in dialysates of striatum during cerebral ischemia (0–120 min) and reperfusion (0–24 h) in rats (n = 6, mean ± standard deviation): **A** Asp, **B** Asn, **C** Glu, **D** Gln, **E** Tau, **F** Gly, **G** GABA, **H** 5-HT, pp *p < 0.05, **p < 0.01 versus vehicle group, values are mean ± SD, n = 6

As shown in Fig. 3, the level of Asp, asparagine (Asn), and glutamine (Gln) in CrCi-CDs groups (409.57 μg/g ± 287.40 μg/g, 12.68 μg/g ± 12.75 μg/g, and 259.3 μg/g ± 132.95 μg/g) was significantly lower than in the vehicle group (997.14 μg/g ± 127.90 μg/g, 43.93 μg/g ± 7.75 μg/g, and 788.9 ± 141.55 μg/g). These levels of inhibitory neurotransmitters such as taurine (Tau) and glycine (Gly) in CrCi-CDs groups were also significantly reduced than in the vehicle group (728.64 μg/g ± 92.28 μg/g, 181.98 μg/g ± 15.2 μg/g). While 5-HT in CrCi-CDs groups (183.09 μg/g ± 73.16 μg/g) increased significantly than it in the vehicle group (110.14 ± 51.36 μg/g).

### The sedative effect of CrCi-CDs

**Fig. 4:**
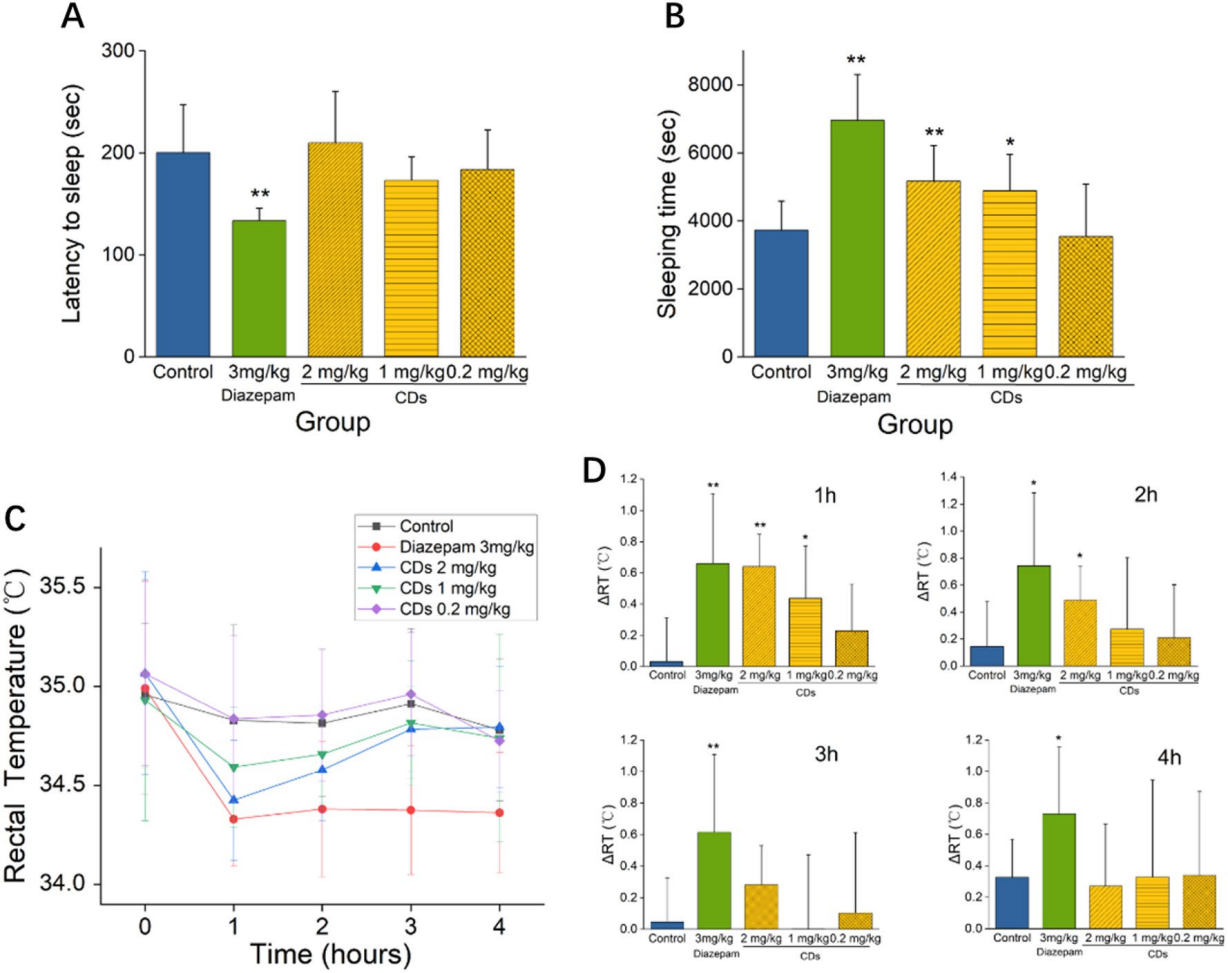
Sedative effects CrCi-CDs. **A** Effects on latency to loss of righting reflex. **B** Total sleep time. Inset, *p < 0.05 and **< 0.01, values are means, n = 10. Interval between administrations of pentobarbital until loss of righting reflex was recorded as onset of sleep. Time from loss to regaining of righting reflex was recorded as duration of sleep. **C** Rectal temperature change of mice treated with CrCi-CDs values are means, n = 10. **D** Rectal temperatures of different groups measured hourly from treatment initiation. Change (Δ) in rectal temperatures (ΔRT) of different groups measured 1, 2, 3, and 4 h after treatment; inset, *p < 0.05 and **p < 0.01

Mice administered pentobarbital (45 mg/kg intraperitoneally, i.p.) showed a loss of the righting reflex within 2–5 min of the treatment. The CrCi-CDs can reduce the pentobarbital-induced sleep latency time from (200.20 ± 47.11) seconds in the control group to (173.20 ± 22.96) seconds (1 mg/kg prepared CrCi-CDs) (Fig. 4A). However, there was no significant difference between the CDs groups and control group. The reference drug, diazepam (3 mg/kg), can significantly (p < 0.01) reduced the latency to sleep.

The middle and high doses of CrCi-CDs groups significantly increased the duration of the pentobarbital-induced sleep compared with control rats (p < 0.01 and p < 0.05, respectively). In comparison, the low dosage CrCi-CDs cannot increase sleeping time. Although the CDs has no effects on sleep latency time, these results of sleeping time suggest CrCi-CDs had sedative properties (Fig. 4B).

The rectal temperature of the mice in each group was recorded at 0, 1, 2, 3, and 4 h after injection (Fig. 4C). The mean rectal temperature before injection was from (34.93 ± 0.61) to (35.07 ± 0.51) °C, and there was no significant difference in all groups. Mice treated with 2 mg/kg CrCi-CDs showed a pronounced rectal temperature reduction (p < 0.01) from (35.07 ± 0.51) to (34.43 ± 0.30) °C. Mice treated with 1 mg/kg CrCi-CDs also exhibited rectal temperature reduction (p < 0.05) from (34.93 ± 0.61) to (34.59 ± 0.30) °C. As shown in Fig. 6D, the Δ rectal temperature (ΔRT) of mice with 2 mg/kg CrCi-CDs was (0.64 ± 0.21) °C, which was not statistically significant compared with the diazepam-treated group (0.66 ± 0.45) °C (Fig. 4D).

While the rectal temperature reduction by these two doses was comparable (p > 0.05), 2 mg/kg CrCi-CDs was significantly superior to the 1 mg/kg dose. Nevertheless, the rectal temperature following treatment with 1 and 2 mg/kg CrCi-CDs extract was shown to slowly rise after 1 h. Furthermore, the temperatures attained the same level with the control group 3 h to 4 h after the injection. In contrast, the positive control, diazepam (3 mg/kg) did not recover the rectal temperature compared to that reported for the CrCi-CDs. The rectal temperature of mice administered 0.2 mg/kg of CrCi-CDs had no statistical significance compared with the values reported for the control group.

Our results suggest that compared with diazepam, CrCi-CDs had the same effect on the rectal temperature within 1 h after injection but induced a faster recovery. The effects of CrCi-CDs extract on the sleeping time suggest it had sedative properties; however, these effects were weaker than those of diazepam, the positive control were. This may be due to the differences in metabolism or mechanisms between CrCi-CDs and diazepam.

### Transcriptome analyses

**Fig. 5:**
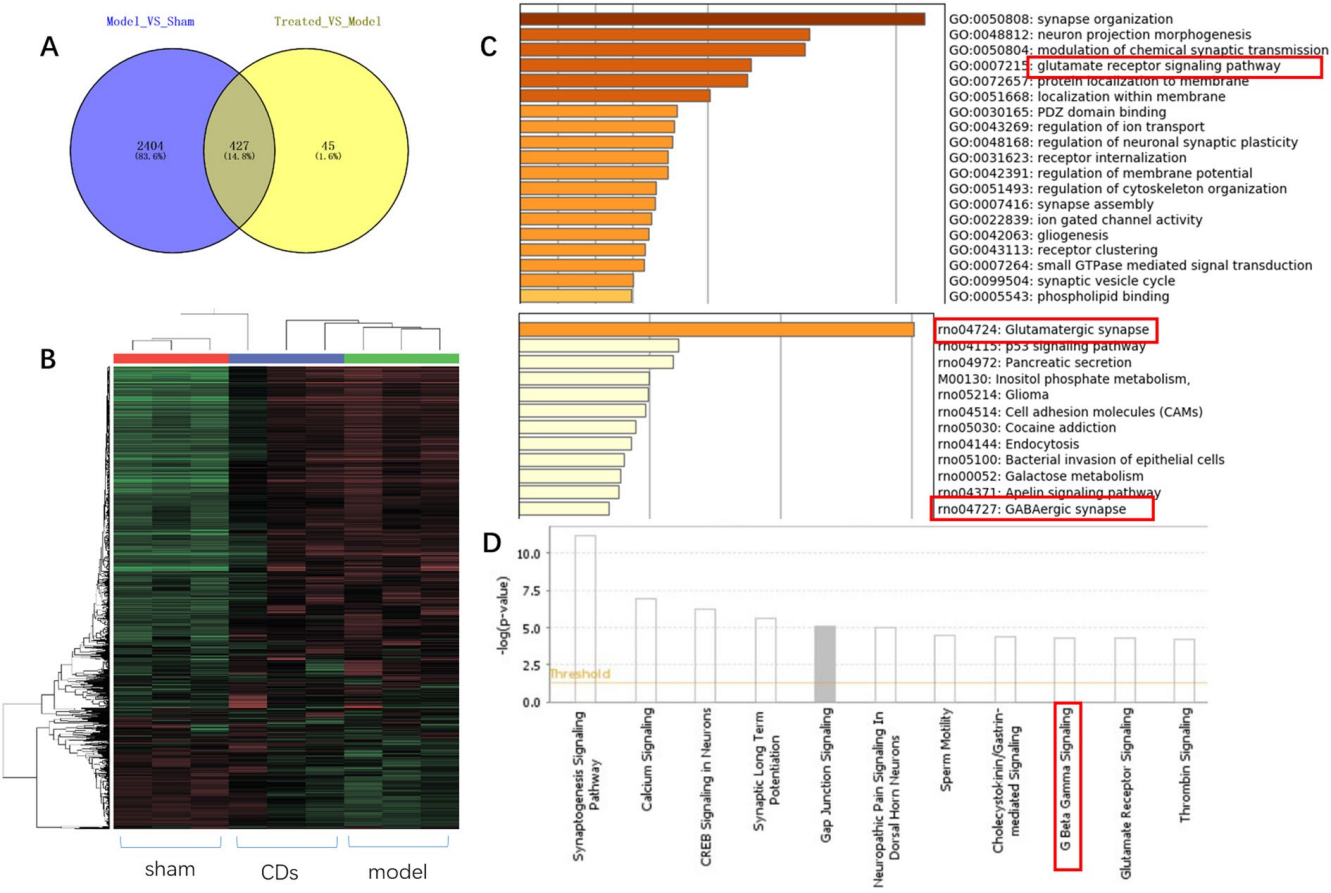
**A** Venn diagram of CrCi-CDs treated brain, **B** GO and META enrichment analysis of significantly changed genes in CrCi-CDs treated brain. **C** Heat map of all differentially expressed genes (n = 3 biologically independent samples for each condition). **D** IPA enrichment analysis of signature gene expression for genes that are related to the neurotransmitters

To discover the molecular mechanisms of CrCi-CDs neuroprotective effect, RNA-Seqencing (RNA-seq) was conducted using nerve cells from control, model and CrCi-CDs-treated mice 24 h after operation. To uncover major molecular changes, Gene Ontology (GO), metascope and IPA enrichment analysis were carried out to evaluate the significantly altered expression genes. Collectively, we identified changes in several pathways that are associated with the excitatory and inhibitory neurotransmitters which may be involved in reducing the neuroexcitatory toxicity such as GLU receptor, including Nrsn2 (highlighted in Fig. 5). In addition, genes that are involved in nerve regeneration, including CREB3(cAMP response element-binding protein) and Serf2, were upregulated (Fig. 5). These data suggest that CDs down-regulate the neural excitation to release the inflammation.

### Toxicity assessment

#### Cell viability assay

**Fig. 6:**
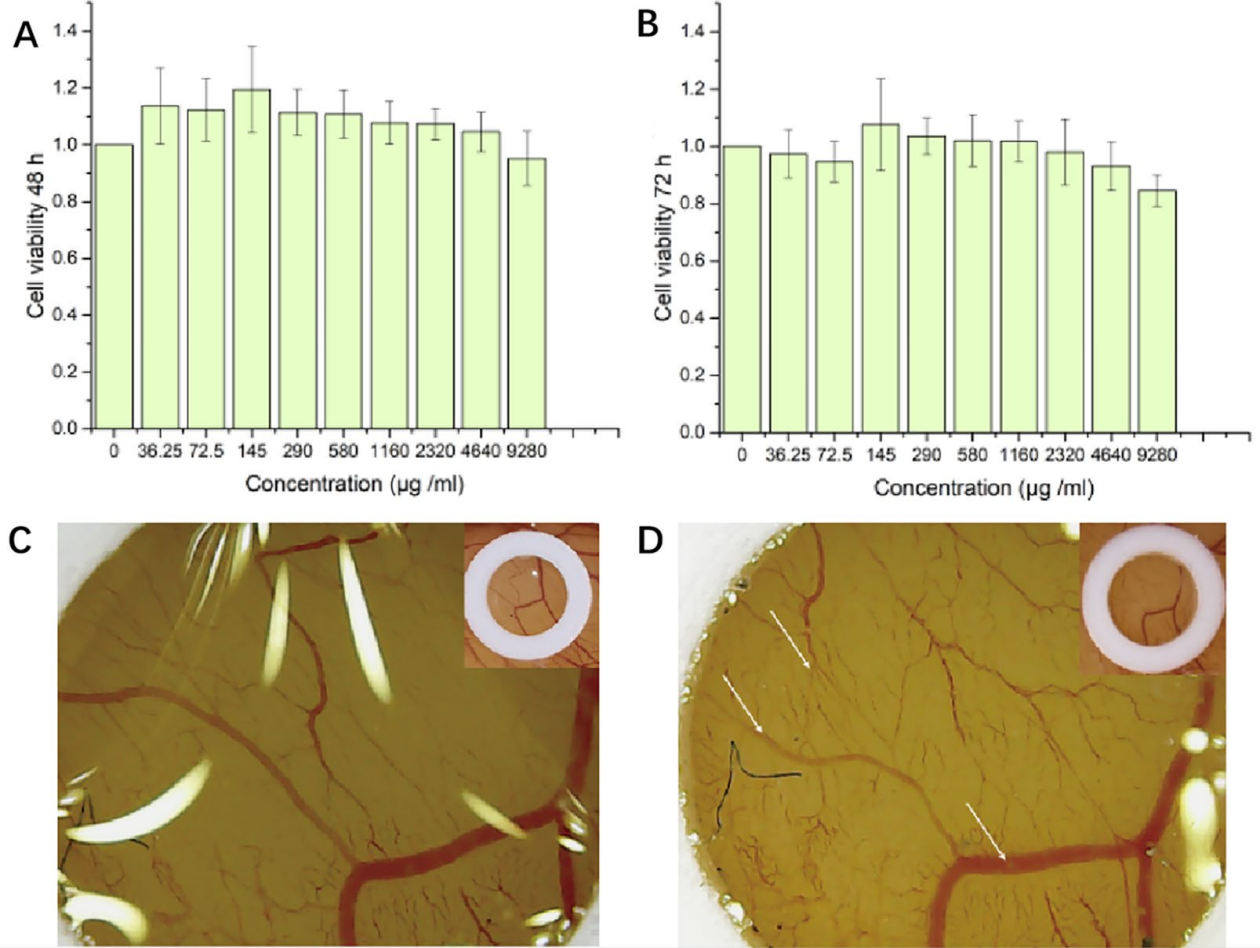
Cell viability of RAW 264.7 after incubation with various concentrations of CrCi-CDs for 48 h (**A**), 72 h (**B**). **C**, **D** Chorioallantoic vascular test of chicken embryo

The potential toxicity of nanoparticles has always been concern in their biological application. As CrCi-CDs is a natural antigen, mouse mononuclear macrophage cell line RAW 264.7 was applied to evaluate the cytotoxicity of CrCi-CDs. The standard CCK-8 assay results were shown in Fig. 6A, B, the cell viabilities of RAW 264.7 cells treated with CrCi-CDs in the concentrations ranging from 36.25 to 9280 µg/mL for 48 h and 72 h. The CrCi-CDs promoted RAW 264.7 cell growth at most concentrations for 48 h. The viability decreased with increasing CrCi-CDs concentration from 4640 to 9280 µg/mL for 72 h. These results indicated that in vitro cytotoxicity of CrCi-CDs was negligible.

#### Chick chorioallantoic membrane assay

After incubation, no obvious bleeding, congestion and coagulation of blood vessels were observed in the CrCi-CDs treated chick chorioallantoic membrane.

## Discussion

In this study, CrCi-CDs pretreatment decreased the EB extravasation in the ischemic hemisphere, suggesting that CrCi-CDs diminish BBB damage caused by cerebral ischemia–reperfusion. To explore the mechanism of CrCi-CDs, the primary concern is whether the nanoscale particles can pass through the blood brain barrier.

Many therapeutic drugs cannot penetrate into the complete (BBB and enter brain tissues within the effective time window [30]). However, cerebral ischemia–reperfusion injury caused increased cerebral vascular permeability, which means the nanoparticles may crosses the blood–brain barrier. Although the nanoparticles were not found in the electron microscope of MCAO rat, we tend to believe the nanoparticles may access into brain in a MCAO model. Similarly, the intraperitoneal injection of GO have been found a neuron-protective effect in previous reports [19, 20].

From these results, we observed that the level of excitatory neurotransmitters (Glu and Asp) reduced significantly, which is believed to play an important role in apoptosis and cell death after cerebral ischemia [31]. The abnormal and excess release of Glutamic acid as well as high-frequency stimulation to the postsynaptic receptor induce excitatory neural toxicity, resulting in the death of neurons. Hence, CrCi-CD is an efficient prevention and cure measures to interrupt the neurotoxicities induced by the over-activated neuron. The RNA-seq results also verified the CDs inhibited the nerve excitation.

Cerebral ischemia–reperfusion injury is a complex pathophysiological process. It is well known that inflammation response plays key roles in ischemia-induced nerve injury. BBB dysfunction can cause neuroinflammation and inflammatory cytokines release, which promotes BBB damage [32]. Inflammatory cascade along with BBB is related to elevated levels of tumor necrosis TNF-α and IL-6 [33], causing brain injury, peaked at 24 h after ischemia in the central nervous system (CNS) [34]. The ischemia–reperfusion injury, nerve necrosis caused by over excitation and followed extensive inflammation leads to disruption of the BBB vasogenic brain edema, cerebral tissue damage and even hemorrhagic transformation triggers more inflammation and sets up a vicious circle.

We also observed that the level of TNF-α and IL-6 in rats were significantly reduced after treated with CrCi-CDs. Obviously, the decrease in nerve excitability reduced inflammation caused by brain edema, which is beneficial to enhancing the tolerance of ischemia and break the wretched cycle.

We further demonstrated the sedative effect of CrCi-CDs by inhibiting excitatory neurotransmitters using the classic pentobarbital sodium induced sleep experiment in mice. This study demonstrate the significant sedative effect of CrCi-CDs and has a tendency to prolong the sleep time of pentobarbital sodium induced mice. Since pentobarbital sodium exerts sedative and hypnotic effects by inhibiting the ascending activation system of brain stem reticulate structure, the results that CrCi-CDs can prolong sleep time indicate that this nanometer component has a certain synergistic sedative and hypnotic effect of pentobarbital sodium.

In the treatment of stroke, clinicians believe hypothermia could reduce brain damage in stroke patients, they used ice to make the body temperature to 34 °C for decreasing body metabolism and inflammation. They also use sedative such as pentobarbital sodium to reach this goal. Perhaps, CrCi-CDs may play its part in reducing neuroexcitatory toxicity which may be a promising strategy for brain protection.

These results of RNA-seq indicated that excitatory and inhibitory neurotransmitters regulation maybe the cause of the CrCi-CDs effect. Similarly, some studies found that single-walled carbon nanotubes can regulate the neural behavior by facilitating dopamine oxidation [15]. However, the specific regulatory mechanism of is not clear. Recently, GO-neurotransmitters interaction studies came to the opposite conclusion. GO was considered to decrease neurotransmitters and pose a potential neurotoxicity [35], while GO was found to reduce oxidative stress and inhibit neurotoxicity [18]. Therefore, The nano–bio interactions may shift the fate of CDs between toxicity and therapeutic potential. A focused investigation of cabon-based nanoparticles medical application is required.

Some indirect effect also should be taken in consideration such as changing the gut microbiome which can secrete neurotransmitters. As intestinal flora regulation often takes a long time, while the CDs takes effect within 24 h, which indicate it maybe not related to intestinal flora. Some researches also found exosome may transfer the mRNA or nanoparticle by passing biological barriers [36–38]. According to current results, these possibilities cannot be ruled out.

The mechanism of ischemia–reperfusion injury is extremely complex. Briefly, cerebral ischemia reperfusion results to inflammatory, edema causing nervous hyperexcitability and aggravating neurological death and massive inflammation. CrCi-CDs can promote inhibitory neurotransmitters release and depletion (or absorbing) excitatory neurotransmitters, which leads to a series of adaptive changes including decreasing in nerve excitability, interrupting the neurotoxicities induced by the over-activated neuron.

Although some hydroxy-rich synthetic CDs were exhibited radical scavenging abilities [18]. We tend to believe the inflammation and ROS decrease were contributed by CDs mediated sedative effect which break the wretched cycle. Nowadays, the impact of nanoparticles on several signaling pathways have been report [19, 20, 34, 35], but the mechanism by which structure and the function of CDs are linked together has not been completely elucidated. According to former researches, dynamic regulation of intracellular ROS seems to be critical to influence the fate of cells. According to our research and these references mentioned, we assume that the neuroprotective effect of CrCi-CDs may be associated with the reduction of neuro- excitotoxicity and inflammatory. And the activity of neuron may be regulated by factors secreted by the astrocyte and microglial cell internalized CDs.

Therefore, in the following work, at least three aspects of work need to be carried out. (1) The large precursor compositions may lead to heterogeneity in natural products-derived CDs and a focused investigation of separation and purification is required. (2) The nano–bio interactions may shift the fate of CDs between toxicity and therapeutic potential. The in vivo distribution of CrCi-CDs requires additional research for a better understanding of CrCi-CDs/biological barrier interactions (especially the blood–brain barrier). (3) According to the CDs biodistribution, explore the impact of nanoparticles on several signaling pathways such as ROS, lysosomal phagocytosis and mTOR pathway.

At present, the stroke treatment strategy usually contains thrombolysis, dilatation and neuron protection. Thrombolytic therapy is an effective method to treat ischemic stroke. However, reperfusion injury is inevitable with the onset of thrombolytic therapy. There are still no effective drugs for reperfusion injury. At present, the stroke treatment strategy usually contains thrombolysis, dilatation and neuron protection. This work provide evidence that the CrCi-CDs derived from *Crinis Carbonisatus* may have the neuroprotective effects on cerebral ischemia–reperfusion injury and therefore may be potentially suitable in clinical therapy on the acute apoplexy cases in combination with thrombolytic drugs.

## Materials and methods

### Chemicals

Dialysis membranes with a molecular weight of 1000 Da were purchased from Beijing Ruida Henghui Technology Development Co., Ltd. (Beijing, China). Chloral hydrate was purchased from Beijing Solarbio Science & Technology Co., Ltd. (Beijing, China). The cell counting kit (CCK-8) and other analytical grade chemical reagents were obtained from Beijing BioDee Biotechnology Co. Ltd. (Beijing, China). All the experiments were performed using deionized water (DW).

### Animals

Animal studies were performed in accordance with the Guide for the Care and Use of Laboratory Animals that was approved by the Committee of Ethics of Animal Experimentation of the Beijing University of Chinese Medicine. Male Sprague–Dawley (SD) rats (weighing 210.0 ± 10.0 g) were purchased from the Laboratory Animal Center, Vital River Laboratory Animal Technology Company with a Laboratory Animal Certificate of Conformity. These animals were housed under the following conditions: temperature, (24.0 ± 1.0) °C; relative humidity, 55–65%, and a 12-h light/dark cycle, with ad libitum access to food and water.

### Synthesis of CrCi-CDs

**Fig. 7:**
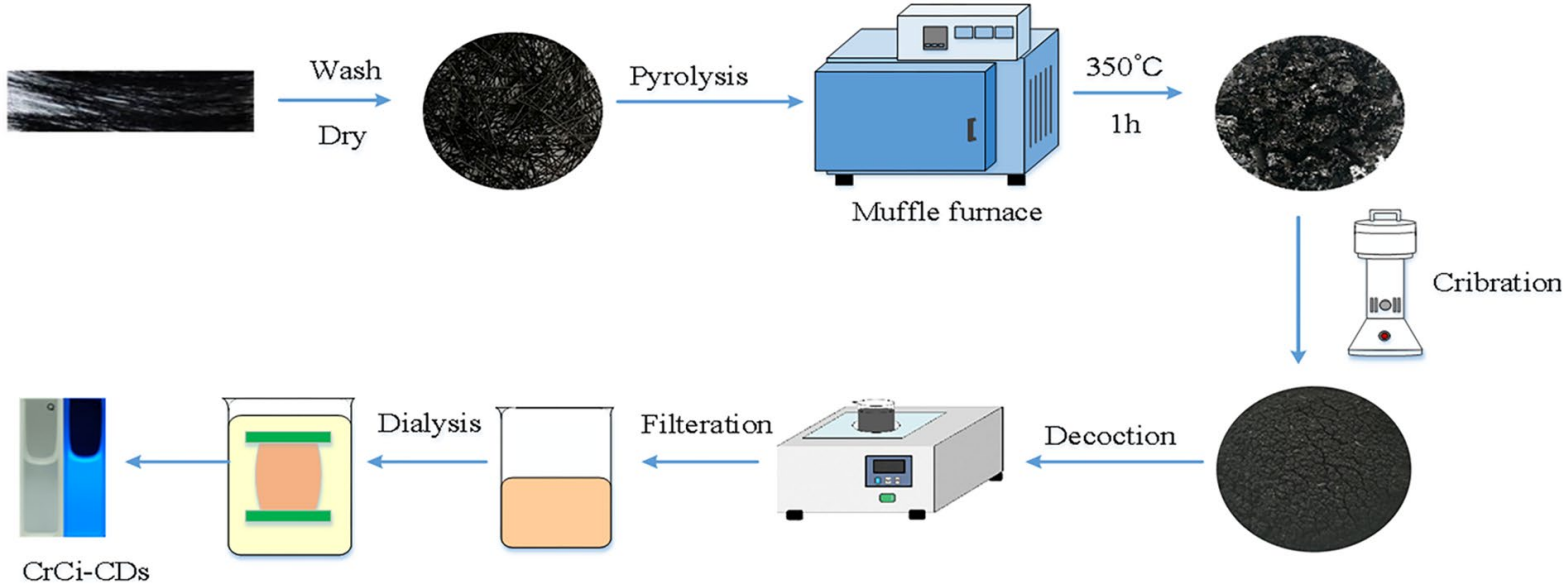
The flowchart for the preparation process of carbon dots derived from Xue-yu-tan (CrCi-CDs)

Briefly, the CrCi was prepared using the traditional processing methods of carbonization and using human hair as raw materials. Hair was collected from healthy volunteers, aged 20–40, which had black straight hair. First, hair was washed with distilled water (DW) to remove impurities, and then with carbonate buffer solution (CBS) to remove grease. After that, the hair was dried in oven at 60 °C for 24 h. The dried hair was calcined by a muffle furnace (TL0612 muffle furnace; Beijing Zhong Ke Aobo Technology Co, Ltd; Beijing, China). The calcined temperature was raised to 350 °C within 1 h and keeping at 350 °C for 1 h. After the temperature cooled to room temperature, the CrCi was crushed with micromill. The fine CrCi powder was soaked in deionized water heating with digital thermostatic water bath pan HH-1 (KY, China) at 100 °C thrice for 1 h each time. The solution obtained was filtered through a 0.22 μm cellulose acetate membrane, and subsequently was dialyzed using a 1000-Da dialysis membrane against DW for 72 h to obtain the CrCi-CDs. The preparation process for the CrCi-CDs is shown in Fig. 7.

### Sample characterization

Photoluminescence experiments were conducted with a Shimadzu RF5-5301 PC spectrofluorimeter (Shimadzu, Japan). UV–vis absorption spectra were obtained using a TU-1991 UV–vis spectrophotometer. Fourier transform infrared spectroscopy (FTIR) was measured in the range of 500–4000 cm^−1^ using a Nicolet 6700 FTIR spectrophotometer. Transmission electron microscopy analyses to study morphology and mean diameter of the resultant samples were carried out on a JEM-2100F (FEI, USA), operating at an accelerating voltage of 200 kV.

### Experimental protocol

**Fig. 8:**
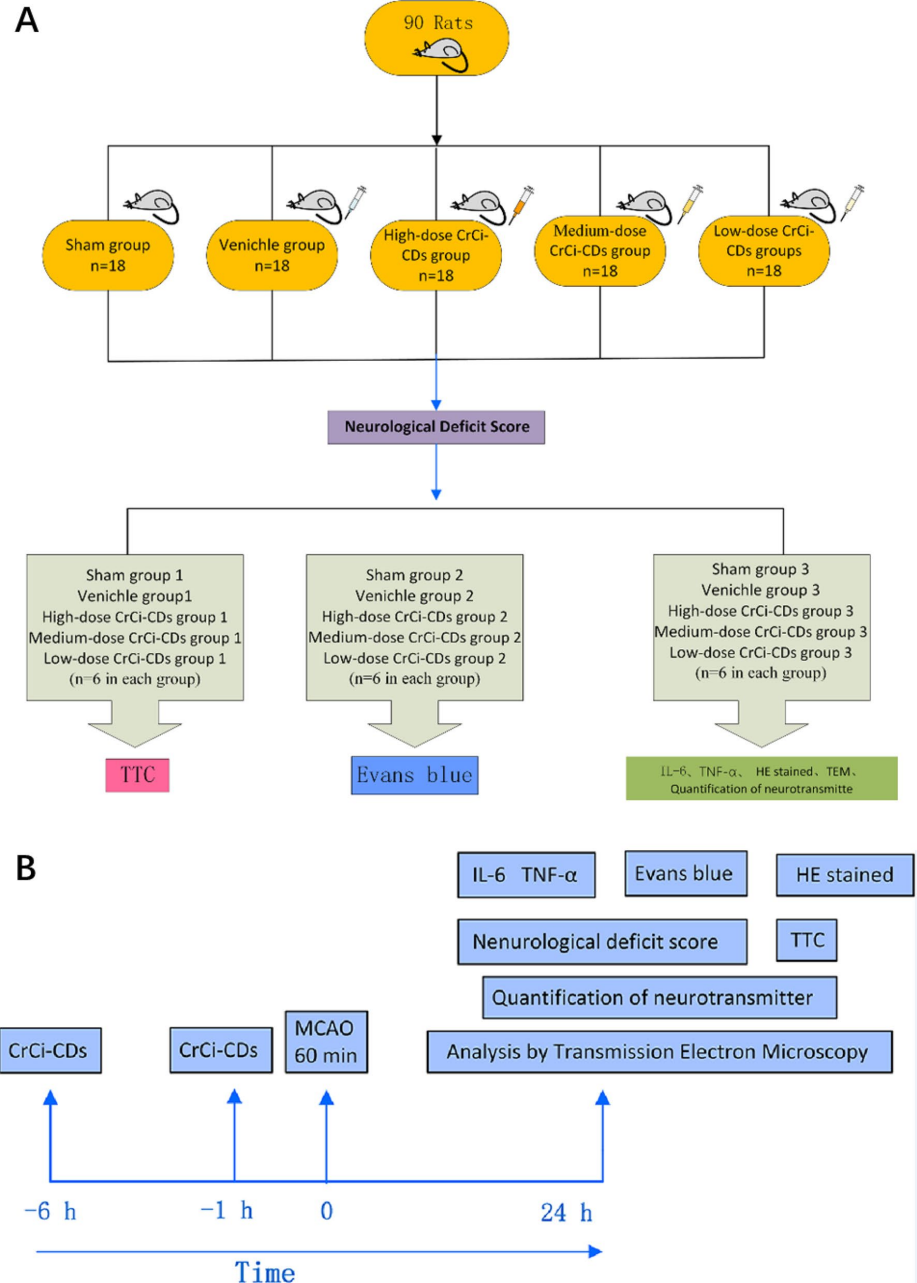
Diagram of experimental protocol

A total of 90 Sprague–Dawley (SD) rats were randomly divided into the following five groups (n = 18 in each group) and the administration of drug was as follows: Sham group (rats received surgery without MCAO); vehicle group (normal saline [NS]); high-, medium- and low-dose CrCi-CDs pretreatment groups (4, 2, and 1 mg/kg, respectively), as shown in Fig. 8. Rats in the sham group were subjected to the same procedure except for the suture insertion. Rats in the vehicle group were injected with saline intraperitoneally, 6 h and 1 h prior to MCAO. Rats in the CrCi-CDs pretreatment groups were pretreated with CrCi-CDs intraperitoneally, at doses of 1, 2, and 4 mg/kg, respectively, 6 h and 1 h prior to MCAO. Rats in vehicle group and CrCi-CDs pretreatment group underwent MCAO for 60 min followed by reperfusion. Rats in sham group underwent the same surgical procedures, except that the filament was not inserted. Twenty-four hours after reperfusion, a grading scale of 0–4 is used to assess behavioral deficits. Then each of these groups was randomly divided into three subgroups, the first subgroup was assessed for BBB permeability, the brain infarction area of rats was determined with TTC staining in the second subgroup, the levels of IL-6 and TNF-α were measured by ELISA, the content of Asp, Glu, 5-HT, Tau, Gly, and GABA in the cerebral cortex were by liquid chromatography–mass spectrometry, and the histological and ultrastructural changes of brain tissue were observed by HE staining and TEM in the third subgroup. As shown in Fig. 8.

### MCAO model

MCAO models were established according to previous methods [25]. Briefly, the animals were anesthetized with chloral hydrate (10%, 3.5 mL/kg, i.p.), and were fixed on rat board. The right common (CCA), internal (ICA) and external (ECA) carotid arteries in rats were isolated and exposed via a midline cervical incision. A filament (tip diameter, 0.32 ± 0.02 mm, Cat#2432A4, Beijing Sunbio Biotech Co., Ltd., Beijing, China) was gently inserted into the middle cerebral artery from the ECA to the ICA, and was advanced up to 18–20 mm into the middle cerebral artery (MCA) from carotid bifurcation. After 1 h of ischemia, the filament was removed and blood was reperfused for 24 h.

### Neurological deficit score

The presence of neurological function deficit was measured by Zea-Longa method [25]. After ischemia for 1 h and reperfusion for 24 h, the neurological behavior scores of each group was evaluated by a 0- to 4-point scale as follows: 0, no neurological dysfunction; 1, failure to extend left forelimb fully; 2, circling to the contralateral side; 3, falling to the left; 4, unable to crawl spontaneously or consciousness disorders. The modified mNSS test [26] was measured at 24 h after MCAO, involving a series of motor, sensory, reflex, and balance measurements.

### Measurement of infarct volume

Ischemic brain damage was assessed by staining brain sections with 2,3,5-triphenyltetrazolium chloride (TTC). Briefly, after 60 min of MCAO and 24 h of reperfusion, rats were anesthetized, the brain tissue was put into the brain groove, and sectioned coronally at a thickness of 2 mm from the front pole to the back coronal, and a total of 5 pieces were cut, and incubated in a solution containing 2%TTC, at 37 °C for 30 min, then fixed in 4% paraformaldehyde overnight, and scanned into a computer. All brain slices were analyzed for the infarct volume using the Image-Pro Plus 6. Lesion volumes were calculated using the following formula [27]: percentage of corrected infarct volume = [(contralateral hemisphere area-(ipsilateral hemisphere-measured infarct area))/contralateral hemisphere area] × 100%.

### Assessing of BBB permeability

Rats were anesthetized again with 10% chloral hydrate 24 h after reperfusion. The rats were injected with 2% EB normal saline solution (4 mL/kg) through a femoral vein for 1 h. Then the rats were thoracotomized and perfused with normal saline through left ventricle until the fluid flowed out of right atrium was colorless. The ipsilateral hemispheres were collected, weighed, homogenated, and were incubated in formamide (1 mL/100 mg) at 60 °C for 48 h, and then centrifuged at a speed of 10,000 r/min for 15 min, the supernatant was collected, fluorescence was measured at 630 nm to evaluate concentration of Evans blue dye. Data are represented as concentration of Evans blue dye (µg)/tissue weight (g).

### HE stain

At 24 h after reperfusion, rats were perfused transcardially first with 0.9% saline and then with ice-cold 4% paraformaldehyde. Paraffin sections of rat brain tissue were soaked and washed with xylene twice, 5 min each time. Gradient ethanol (100%, 95%, 90%, 80%, 70%) were used to soak and wash for 3 min each time. HE staining was performed, and the lesion was observed under the microscope and photographed.

### Ultrastructural analysis by transmission electron microscopy

Brains were fixed after heart perfusion with a 2.5% glutaraldehyde solution. The ischemic cortical tissue was taken, with a size of about 1 mm × 1 mm × 3 mm, and fixed in 2.5% glutaraldehyde solution for more than 2 h. The specimens were fixed, dehydrated, soaked, embedded and solidified during the preparation process of conventional electron microscope. Finally, the tissues were observed using TEM.

Rats were sacrificed at 24 h after MCAO, and tissue samples were collected from the ischemic penumbra, following fixation in 2.5% (w/v) glutaraldehyde overnight. The tissues were embedded in Araldite for coronal sections. Finally, the sections of the sample were observed using a Hitachi TEM.

### Enzyme-linked immunosorbent assay (ELISA) for inflammatory cytokines

Brain tissues from the cortex of the ischemic hemisphere were homogenized according to the manufacturer’s recommendations, and after centrifugation, supernatant was collected. The levels of IL-6, TNF-α in cortex were measured by using commercially obtained ELISA kits specifically for IL-6, TNF-α (Uscn Life Science Inc., Wuhan, China). The assay was carried out according to the instructions provided by the manufacturer. The optical density (OD) 450 nm was calculated by subtracting the background, and standard curves were plotted.

### Quantification of neurotransmitter by liquid chromatography–mass spectrometry

Liquid chromatography and mass spectrometry conditions Quantification of polar neurotransmitters and related compounds in brain homogenates was performed using an Water Acquity UPLC I-CLASS system for the chromatographic separation coupled to a triple quadrupole (Xevo TQ-S micro) mass spectrometer provided with an orthogonal Z-spray-electrospray interface (ESI) (Waters Associates, Milford, MA, USA). The drying and nebulizing gas was nitrogen. The desolvation gas flow was set to 800 L/h and the cone gas flow to 10 L/h. A capillary voltage of 1.5 kV was used in positive ionization mode. The nitrogen desolvation temperature was set to 400 ºC and the source temperature to 150 °C. Collision gas was argon and the injection volume was 5 µL. The chromatographic separation was achieved at 50 °C using an ACQUITY UPLC BEH Amide 1.7 micron (2.1 × 100 mm) (Waters Associates). Mobile phase A was ammonium formate 25 mM with formic acid (0.01% v/v) dissolved in mixture acetonitrile:water (1:1). Mobile phase B was ammonium formate 25 mM with formic acid (0.01% v/v) in water.

### Cellular toxicity

CCK-8 was applied to assess cell toxicity of CrCi-CDs [28]. RAW264.7 cells (1 × 10^4^) were seeded into each well of 96 well-plates and grown overnight under a 37 °C and 5% CO_2_. Then the cells were treated with CrCi-CDs at different concentrations, ranging from 36.25 to 9280 μg/mL for 48 h and 72 h, respectively. After washed three times with PBS, DMEM (90 μL) and CCK-8 (10 μL) were subsequently added to each well and incubated for another 1.5 h at 37 °C, and a microplate reader was used to measure the OD 450. The cell viability can be calculated using the following formula:

$$ {\text{Cell}}\;{\text{viability}}\left( {\% \;{\text{of}}\;{\text{control}}} \right) = {{\left( {{\text{ OD}}_{{{\text{test}}}} - {\text{OD}}_{{{\text{blank}}}} } \right)} \mathord{\left/ {\vphantom {{\left( {{\text{ OD}}_{{{\text{test}}}} - {\text{OD}}_{{{\text{blank}}}} } \right)} {\left( {{\text{OD}}_{{{\text{control}}}} - {\text{OD}}_{{{\text{blank}}}} } \right)}}} \right. \kern-\nulldelimiterspace} {\left( {{\text{OD}}_{{{\text{control}}}} - {\text{OD}}_{{{\text{blank}}}} } \right)}}, $$

OD_test_, OD_blank_, and OD_control_ represent the A450 nm of the experimental, blank and control groups, respectively.

### The sedative effect of CrCi-CDs

The sedative effect of this nanometer component was studied by the classic sleep experiment induced by pentobarbital sodium in mice, and the changes of rectal temperature in mice were also detected to explore the sedative mechanism.

#### Pentobarbital-induced sleeping time test

Briefly, pentobarbital induced sleeping time test was performed. Mice were given CDs (2, 1, 0.2 mg/kg, i.p.) 30 min before the injection of pentobarbital (45 mg/kg, i.p.). All drugs, used as standard, were administered intraperitoneally (i.p.). The 0.9% saline (10 mL/kg) was administered by intraperitoneally as the control group. Sleep was induced by the administration of pentobarbital (45 mg/kg, i.p.). The latency of the loss of the righting reflex after pentobarbital administration (“onset” time) and the total sleeping time (the time between the loss and the recovery of the righting reflex, “duration”) were determined for each mouse. The mouse was considered as being awake if it could right itself (return to upright position).

#### The effect on rectal temperature

Rectal temperature was measured by MP150 System (BIOPAC, USA) per rectum on the day of treatment commencement. After the treatment, the rectal temperature of all the mice in each group was recorded at 0 h, 1 h, 2 h, 3 h, and 4 h.

### RNA-seq

A total of 2 ng of RNA from each sample was used to generate RNA-seq libraries using a SMART-Seq v4 Ultra Low Input RNA kit (Takara, 634888) and Nextera XT DNA Library Preparation Kit (Illumina, FC-131-1024). Single-end sequencing reads were obtained using the Illumina NextSeq 500 platform. Sequencing reads from RNA-seq libraries were trimmed using Trim Galore and aligned to the mouse reference genome using STAR aligner59. Gene expression levels were normalized and differential expression of genes was calculated using the DESeq2 package in R60. Gene set functional enrichment analysis was performed using DAVID61, 62.

### Chick chorioallantoic membrane assay

Fertilized eggs were incubated for 24 h at room temperature in an incubator with a temperature of (37 ± 0.5) °C and a relative humidity of (62.5 ± 1.5)%. After incubated to 10 days of age, The eggs with good growth were screened by the egg catcher. And the eggshells at the top of the air chamber were cracked and removed in super clean table. Drop 3–5 drops of normal saline on the eggshell membrane, carefully peel off the eggshell membrane, exposing the allantoic chorionic membrane. Place a sampling ring on the allantoic membrane. 0.3 mL of transparent liquid subjects were directly dripped into the sampling ring with a pipette or a disposable sterile syringe. After incubation at 37° for half an hour, bleeding, congestion and coagulation of blood vessels were observed with a postural microscope, and the stimulation score was calculated.

### Statistical analysis

Statistical analyses were performed using statistical package for the social sciences (SPSS, version 17.0). A value of P < 0.05 was considered statistically significant. The normally distributed data with equal variances were expressed as the mean ± standard deviation. The non-normally distributed data were expressed as the median (quartile range). The infarct volume data were analyzed using the Image-Pro Plus 6.0 software (version 6.00, Image-Pro Plus Software).

## Conclusion

In summary, a new type of carbon dots was identified and purified from a charcoal drug *Crinis Carbonisatus.* Its security was investigated by cell viability assay and chick embryo in vitro. Pharmacodynamic experiments indicated the CrCi-CDs derived from *Crinis Carbonisatus* may have the neuroprotective effects on cerebral ischemia–reperfusion injury, which may correlated to regulate neurotransmitters and reduce neuroexcitatory toxicity.

This work indicated that the sedative effect of CrCi-CDs is associated with the reduction of neuroexcitotoxicity and inflammatory. Hence, the CrCi-CDs have potential value in clinical therapy on the acute apoplexy cases in combination with thrombolytic drugs.

Our results may provide guidance for further studies on the nerve related bioactivities of CDs. Moreover, it also give new insights into potential healthcare applications of CDs. While, Further studies are needed to elucidate how CrCi-CDs exert its protective effects against cerebral ischemia–reperfusion injury and participate in the mechanism of anti-inflammatory response.

## Supplementary Information

**Additional file 1: Table S1.** The comparison with previously published CDs.

## Data Availability

Data sharing is applicable to this article.
