# Supplementary material for: The neuroprotective effect of pretreatment with carbon dots from Crinis Carbonisatus (carbonized human hair) against cerebral ischemia reperfusion injury
Source: J Nanobiotechnology. 2021 Aug 28;19:257. doi: 10.1186/s12951-021-00908-2 (PMC8399708; doi:10.1186/s12951-021-00908-2)
Supplement: Supplementary file 1 — Additional file 1: Table S1. The comparison with previously published CDs. [file 12951_2021_908_MOESM1_ESM.docx]

**Supporting information for**

**The Neuroprotective Effect of Pretreatment with Carbon Dots from *Crinis Carbonisatus* (Carbonized human hair) Against Cerebral Ischemia Reperfusion Injury**

**Table S1 Table S1 The comparison with previously published CDs**

| **Sources** | **Parts** | **Application** | **References** |
| --- | --- | --- | --- |
| Zingiberis rhizoma | Rhizome | Analgesia | 1 |
| Selaginella tamariscina | Stem+leaf | Hemostasis | 2 |
| Paeoniae Radix Alba | Root | Hepatoprotection | 3 |
| mulberry silkworm cocoon | - | Anti-inflammatory | 4 |
| Lonicerae japonicae Flos | Flower | Anti-inflammatory effect/ alleviating fever | 5 |
| Junci Medulla | Medulla | Hemostasis and hepatoprotection | 6 |
| Radix Puerariae | Root | Improve the solubility and bioavailability of baicalin | 7 |
| Aurantii fructus immaturus | Fruit | Anti-gouty effect | 8 |
| Charred Fructus crataegi | Fruit | Maltase and sucrase inhibitory activities | 9 |
| Phellodendri Chinensis Cortex | Cortex | Treatment of *Deinagkistrodon acutus* venom-induced acute kidney injury | 10 |
| Phellodendri Chinensis Cortex | Cortex | Hemostasis | 11 |
| Schizonepetae Spica | Spica | Hemostasis | 12 |
| Cirsium setosum | Aerial parts | Hemostasis | 13 |
| Phellodendri Chinensis Cortex | Cortex | Treatment of psoriasis /immunoregulation | 14 |

**Reference:**

1. Zhang M , Cheng J , Y Zhang, et al. Green synthesis of Zingiberis rhizoma-based carbon dots attenuates chemical and thermal stimulus pain in mice. Nanomedicine, 2020, 15(9),851-869.

2.Zhao, Y., Zhang, Y., Kong, H., Zhang, M., et al. Haemostatic Nanoparticles-Derived Bioactivity of from Selaginella tamariscina Carbonisata. Molecules, (2020). 25(3), 446.

3. Zhao, Y., Zhang, Y., Kong, H., et al. Carbon Dots from Paeoniae Radix Alba Carbonisata: Hepatoprotective Effect. International Journal of Nanomedicine, (2020). 15, 9049–9059.

4. Wang, X., Zhang, Y., Kong, H., Cheng, J., et al.. Novel mulberry silkworm cocoon-derived carbon dots and their anti-inflammatory properties. Artificial Cells Nanomedicine and Biotechnology, (2020). 48(1), 68–76.

5.Wu, J., Zhang, M., Cheng, J., et al. Effect of Lonicerae japonicae Flos Carbonisata-Derived Carbon Dots on Rat Models of Fever and Hypothermia Induced by Lipopolysaccharide. International Journal of Nanomedicine, (2020).15, 4139–4149.

6. Cheng, J., Zhang, M., Sun, Z., et al. Hemostatic and hepatoprotective bioactivity of Junci Medulla Carbonisata-derived Carbon Dots. Nanomedicine: Nanotechnology, Biology and Medicine, (2019).14(4), 431–446.

7. Luo, J., Kong, H., Zhang, M., Novel Carbon Dots-Derived from Radix Puerariae Carbonisata Significantly Improve the Solubility and Bioavailability of Baicalin. Journal of Biomedical Nanotechnology, (2019).15(1), 151–161.

8. Wang, S., Zhang, Y., Kong, H., et al. Antihyperuricemic and Anti-Gouty Arthritis Activities of Aurantii Fructus immaturus Carbonisata-Derived Carbon Dots. Nanomedicine: Nanotechnology, Biology and Medicine, (2019).14(22), 2925–2939.

9 Lu, F., Zhang, Y., Cheng, J., Zhang, M., et al. Maltase and sucrase inhibitory activities and hypoglycemic effects of carbon dots derived from charred Fructus crataegi. Materials Research Express, (2019). 6(12), 125005.

10. Zhang, M., Cheng, J., Sun, Z., et al. Protective Effects of Carbon Dots Derived from Phellodendri Chinensis Cortex Carbonisata against Deinagkistrodon acutus Venom-Induced Acute Kidney Injury. Nanoscale Research Letters, (2019).14(1), 377–377.

11. Liu, X., Wang, Y., Yan, X., et al. Novel Phellodendri Cortex (Huang Bo)-derived carbon dots and their hemostatic effect. Nanomedicine: Nanotechnology, Biology and Medicine, (2018).13(4), 391–405.

12.Sun, Z., Lu, F., Cheng, J.,et al. Haemostatic bioactivity of novel Schizonepetae Spica Carbonisata-derived carbon dots via platelet counts elevation. Artificial Cells Nanomedicine and Biotechnology, (2018). 46.

13.Luo, J., Zhang, M., Cheng, J., et al. Hemostatic effect of novel carbon dots derived from Cirsium setosum Carbonisata. RSC Advances, (2018).8(66), 37707–37714.

14. Zhang, M., Cheng, J., Hu, J., et al. Green Phellodendri Chinensis Cortex-based carbon dots for ameliorating imiquimod-induced psoriasis-like inflammation in mice. Journal of Nanobiotechnology, (2021). 19(1), 105.
